# A Novel Nitinol Spherical Occlusion Device for Liver Cancer

**DOI:** 10.3390/ma9010019

**Published:** 2016-01-02

**Authors:** Hao-Ming Hsiao, Yi-Ping Wang, Chun-Yi Ko, Yu-Han Cheng, Han-Yu Lee

**Affiliations:** Department of Mechanical Engineering, National Taiwan University, Taipei 10617, Taiwan; r03522820@ntu.edu.tw (Y.-P.W.); r03522807@ntu.edu.tw (C.-Y.K.); r04522805@ntu.edu.tw (Y.-H.C.); b01502020@ntu.edu.tw (H.-Y.L.)

**Keywords:** liver cancer, hepatic artery embolization, spherical occlusion device, nitinol alloy

## Abstract

Liver cancer or hepatic cancer is a cancer that originates in the liver. It is formed from either the liver itself or from structures within the liver, including blood vessels or the bile duct. Liver cancer can be a life-threatening condition, but it may be cured if found early. Hepatic artery embolization is one of the treatment options involving the injection of substances to reduce the blood flow to cancer cells in the livers of patients with tumors that cannot be removed by surgery; however, this treatment has some limitations. In this paper, we propose a novel nitinol “spherical occlusion device” concept, the first of its kind in the world. Our proposed spherical occlusion device is able to reduce the blood flow to cancer cells by deploying it in the upstream hepatic artery supplying blood to the liver. Moreover, it could carry multiple chemotherapy or radioactive drugs for delivery directly to the target site. Nitinol alloy was chosen as the device material due to its excellent super-elastic property. Computational models were developed to predict the mechanical response of the device during manufacturing and deployment procedures, as well as its hemodynamic behavior. Simulation results showed that the presence of the spherical occlusion device with 14%–27% metal density deployed at the upstream location of the right hepatic artery had significant occlusion effects, with the average blood flow rate cut down by 30%–50%. A pulsed fiber laser and a series of expansions and heat treatments were developed to make the first prototype of the spherical occlusion device for the demonstration of our novel concept.

## 1. Introduction

Liver cancer is the sixth most frequent cancer and the second leading cause of cancer death in the world. More than 700,000 people worldwide are diagnosed with liver cancer each year. In 2012, it occurred in 782,000 people and resulted in 746,000 deaths, a high mortality rate of 95% [1]. The leading cause of liver cancer is cirrhosis due to hepatitis B, hepatitis C, or alcohol. Chronic infection with hepatitis B or hepatitis C virus is associated with a higher risk of developing hepatocellular carcinoma [2,3]. Alcohol abuse represents another cause of cirrhosis and is also a major contributor to hepatocellular carcinoma in many countries, with some evidence of a synergistic effect in the presence of hepatitis B or hepatitis C virus infection [4,5,6].

The treatment options of liver cancer include surgery, radiofrequency ablation, alcohol (ethanol) injection, chemotherapy, and hepatic artery embolization. Surgery is the major treatment option for early-stage liver cancer; however, in only a small fraction of people is liver cancer diagnosed early enough to benefit from surgery. Radiofrequency ablation uses imaging guidance to pass a needle electrode through the skin and into a liver tumor, after which high-frequency electrical currents travel through the electrode to destroy tumor cells. Alcohol injection, which destroys tumors by dehydrating tumor cells, is the most useful approach for patients with a small number of tumors. Chemotherapy uses anti-cancer drugs that are injected into a vein, making this treatment potentially useful for patients in whom cancer has spread to most parts of the body. Hepatic artery embolization injects substances to cut off the blood flow to tumor cells in the liver and thereby reduce the supply of oxygen and food to the cancer.

Hill *et al.* evaluated the clinical performance of Amplatzer vascular plug in patients with congenital heart disease. It was a self-expanding cylindrical or cone device used for embolization in the peripheral vasculature. The plug was made out of nitinol mesh wires with polyester fabric layers that provided a good nidus for clot formation. Results showed that complete vessel occlusion was achieved within 10 min in 94% of patients [7]. Dnenkamp *et al.* investigated the effects of vascular occlusion in mouse tumors by using D-shaped metal clamps in an attempt to induce complete vascular occlusion. Results showed that clamping for the period of 2–8 h induced a progressive delay in tumor growth, while the local tumor control can reach 100% with an occlusion over 24 h [8]. Hafström *et al.* studied the occlusion treatment option of liver metastases from colorectal cancer. Several plastic slings were placed around the selected hepatic artery and tightened for 16 h. Results showed that the mean survival time in the treated group was nine months longer than the control group [9]. These studies clearly demonstrate that vascular occlusion does have a strong influence in delaying tumor growth and can be effective in cancer treatment. Therefore, preventing the blood flow to tumor cells in the liver presents a potential treatment option for liver cancer in the future.

Nitinol, an excellent material for biomedical applications, possesses both super-elastic and shape-memory properties. Due to these unique properties, nitinol is able to first sustain deformation and then spring back to its original configuration after the loading is removed. With proper fixtures and heat-treatment process, nitinol could be shaped into a wide variety of configurations based on the clinical needs. Nitinol also forms a titanium-dioxide layer on its surface after electro-polishing, which not only prevents the generation of nickel compounds but also increases the device resistance to corrosion. Currently, it is widely used in interventional medical devices, with the most well-known application being the self-expanding stent for peripheral artery diseases [10].

In this paper, we propose a novel nitinol “spherical occlusion device” concept, the first of its kind in the world. Our proposed spherical occlusion device is able to reduce the blood flow to tumor cells by deploying it in the upstream hepatic artery supplying blood to the liver. Moreover, it could carry multiple chemotherapy or radioactive drugs for delivery directly to the target site and act as an implantable long-term drug delivery device. Drug efficacy and safety can be improved by delivering drugs specifically to a target site at a controlled release rate, which would help to keep the drug concentration in the local environment within the therapeutic range to avoid toxicity, reduce side effects, and maximize therapeutic effects [11]. Our spherical occlusion device represents a new treatment option for liver cancer. Nitinol alloy was used as the device material due to its excellent self-expanding property. Finite element (FEA) models were developed to predict the mechanical response of the device during manufacturing and deployment procedures, which included a series of expansions and heat treatments to the target size and crimping inside a cylindrical catheter for delivery. A computational fluid dynamics (CFD) model was developed to evaluate the effects of the presence of the spherical occlusion device on the flow rate change. A pulsed fiber laser and a series of expansions and heat treatments were used to make the first prototype of the spherical occlusion device for the demonstration of our novel concept.

## 2. Materials and Methods

### 2.1. Design Concept of Spherical Occlusion Device

There are several plug devices currently available in the market for Patent Ductus Arteriosus (PDA) and Ventricular Septal Defect (VSD) diseases. For example, Vallecilla *et al.* developed a double-cone shaped nitinol device made with nitinol mesh wires and dacron fibers for PDA occlusion [12]. Transcatheter closure for PDA is an established technique today, making itself a potential treatment option for PDA. Both *in vivo* and *in vitro* experiments were conducted and accurate positioning to the target sites was achieved in all cases. *In* *vivo* experiments performed in new born calves showed that total closure was successful and the blood flow did not pass through PDA after implantation. The shape of these commercial plug devices is usually double-cone (dog-bone), cylindrical, or umbrella which has a longer contacting zone with the artery. Since most of the arteries are tapered from the proximal to distal end, this may create some size mismatch issue where the distal section of the artery is over-expanded by the plug and the proximal section is mal-apposed (no contact). Our proposed spherical occlusion device is the first of its kind in the world. Its contact zone with the artery is merely a circumferential ring, potentially minimizing the size mismatch issue mentioned above.

**Figure 1:**
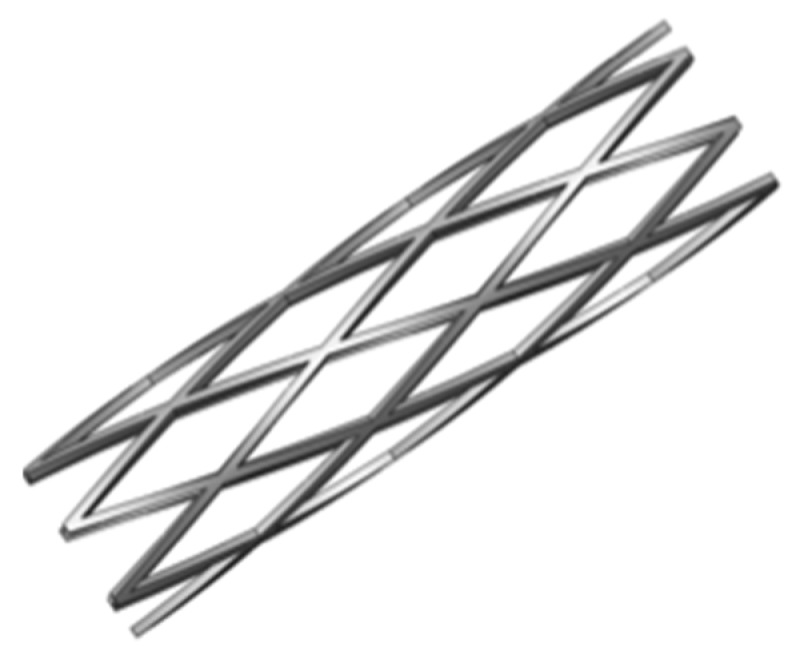
Spherical occlusion device in its initial cylindrical tubing configuration.

When designing a spherical occlusion device, it is critical that the device can be crimped inside a small catheter for delivery. Upon arrival at the target site, it is released from the catheter and self-expands to a sphere of the designed diameter to reduce the blood flow. The expanded diameter of the spherical occlusion device can be slightly larger than that of the hepatic artery to prevent the device from migrating inside the hepatic artery. It is challenging to design a spherical occlusion device because this device has to withstand large deformations that occur during a series of spherical expansions and subsequent crimping. In order to achieve this goal, a parametric design scheme was applied to the design process and integrated with the developed finite element models to assess the device behavior and reduce the development time [13]. With these integrated tools, a modified design was completed and evaluated within much less time than with traditional approaches. A 2D drawing of the spherical occlusion device was first developed and then wrapped around a 3D cylinder using SolidWorks (Dassault Systemes SolidWorks Corp., Waltham, MA, USA). The spherical occlusion device in its initial cylindrical tubing configuration is shown in Figure 1. This cylindrical design will be laser-cut from a small hypotube and then expanded to a sphere by a series of expansions at a later stage. The flow rate (or occlusion rate) can be tailored to a desired level, depending on the density of the spherical occlusion device designed. The device density is defined as:

(1) $$\text{Device density}=\frac{A_{m}}{A_{S}}$$

where *A_m_* is the metal surface area and *A_S_* is the sphere surface area.

### 2.2. Finite Element Models

Computational modeling is a powerful tool in the medical device industry due to its ability to give insights into various aspects of the device performance, which may consequently improve its clinical outcome [14,15,16]. In this study, finite element models were developed to evaluate the mechanical integrity of the spherical occlusion device by the following steps:

Step 1: Expand the device to spheres of 2.0, 3.0, 4.0 and 5.2 mm in diameter and anneal it after each expansion to relieve residual stresses.
Step 2: Crimp the spherical occlusion device inside a cylindrical catheter with an inner diameter of 1.6 mm for delivery.
Step 3: Release the spherical occlusion device inside an artery with a diameter of 5.2 mm.

**Figure 2:**
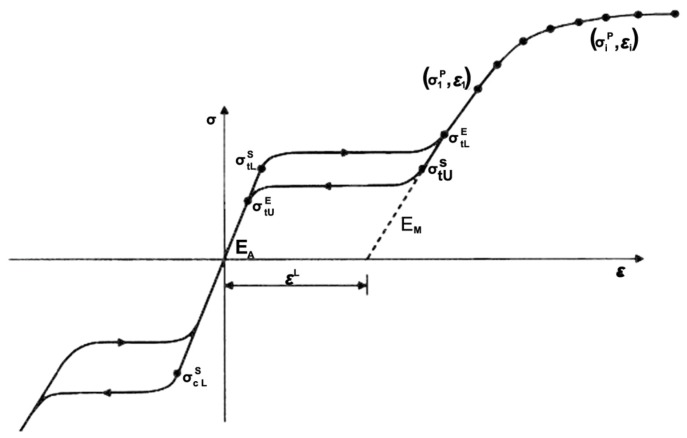
Nitinol stress-strain curve and the corresponding data inputs for the ABAQUS UMAT subroutine.

Finite element analysis was employed using the ABAQUS/Standard finite element solver (Dassault Systemes Simulia Corp., Providence, RI, USA). When the ABAQUS UMAT (User-defined Material) subroutine is used, data points representing different stages of the nitinol stress-strain curve can be input into the FEA model to fully characterize its behavior [17]. Figure 2 shows the nitinol stress-strain curve during loading/unloading and the corresponding data inputs for the ABAQUS UMAT subroutine. In order to simulate multiple expansions and heat treatments during manufacture and subsequent crimping inside a catheter, a sphere with a diameter of 1.5 mm and a cylinder with a diameter of 10.0 mm were built into the model, with the sphere and cylinder placed inside and outside the spherical occlusion device, respectively. During each expansion and its subsequent heat treatment, the inner sphere was expanded to the target diameter and the stress-strain values in all elements of the device were re-set to zero to simulate the stress relief. The deformed geometry of the device was extracted for the next expansion until the final diameter was reached. After completing the final expansion, the outer cylinder was used to compress the device to the catheter size to simulate crimping of the device inside a catheter. For the element selection, the device body was assigned with the incompatible mode eight-node brick element (C3D8I), while the inner sphere and outer cylinder were assigned with the reduced integration four-node quadrilateral surface element (SFM3D4R).

### 2.3. Computational Fluid Dynamics Model

#### 2.3.1. Governing Equations

The blood flow behavior was described by the conservation of mass and momentum, as given in Equations (1) and (2), respectively:

(2) ∇⋅ν=0

(3) $$\rho \left(\frac{\partial \nu }{\partial t}+\nu \cdot \nabla \nu \right)=-\nabla p+\mu \nabla^{2}\nu +f$$

**Figure 3:**
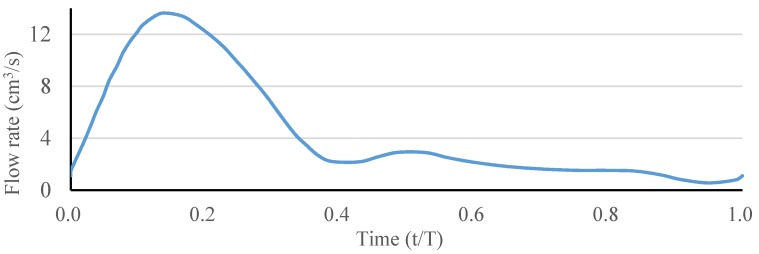
Waveform of the inlet blood flow rate in a single pulse.

The blood flow was assumed to be incompressible and laminar, and its density to be 1060 kg/m^3^. The ANSYS FLUENT software (ANSYS, Inc., Canonsburg, PA, USA) was used to solve the above governing equations and provide robust solutions by applying the pressure–velocity coupled algorithm, which is able to solve the velocity and pressure fields simultaneously. The artery wall was assumed to be a no-slip boundary condition. A fully developed pulsatile flow was prescribed as the inlet boundary condition, and the uniform pressure was prescribed as the outlet boundary condition. The waveform of the inlet blood flow rate in a single pulse is shown in Figure 3. The Carreau model was used to describe the non-Newtonian behavior of the blood flow as follows:

(4) $$\mu =\mu_{\infty }+\left(\mu_{0}-\mu_{\infty }\right){\left[1+{\left(\lambda \dot{\gamma }\right)}^{2}\right]}^{\frac{n-1}{2}}$$

where $\mu_{\infty }$ is viscosity at infinite shear rate, $\mu_{0}$ is viscosity at zero shear rate, λ is relaxation time, and *n* is power index related to material. In this study, these parameters were obtained by fitting the Carreau model to the experimental data [18,19,20], where the fitted values were $\mu_{\infty }$ = 0.0035 kg/ms, μ_0_ = 0.25 kg/ms, λ = 25.0 s, and *n* = 0.25.

#### 2.3.2. Hepatic Artery Bifurcation Model

**Figure 4:**
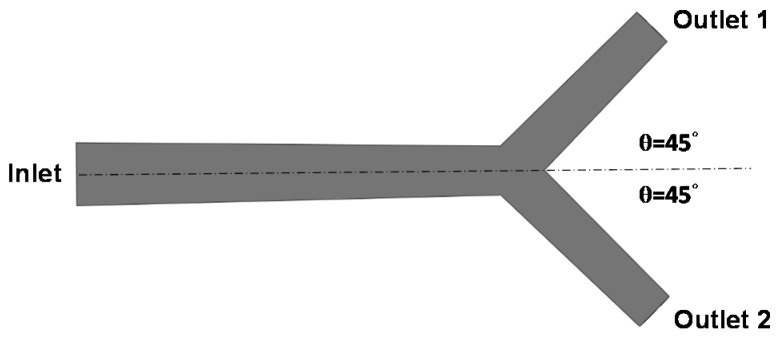
Hepatic artery bifurcation model for the computational fluid dynamics (CFD) simulation.

A simple hepatic artery bifurcation model was constructed to evaluate the effects of the presence of the spherical occlusion device on the flow rate change (Figure 4). This bifurcation model represents a common hepatic artery branching into smaller right and left hepatic arteries. The diameter of the common hepatic artery (inlet) was 6 mm, while the diameters of the right and left hepatic arteries (outlets 1 and 2) were both 4 mm. The bifurcation angle between outlets 1 and 2 was assumed to be 90°, with each outlet branch at a 45° angle to the centerline of the inlet. The spherical occlusion device was placed in the upstream location of outlet 2 near the branch, with two different occlusion densities evaluated for the flow change.

### 2.4. Laser Cutting

**Figure 5:**
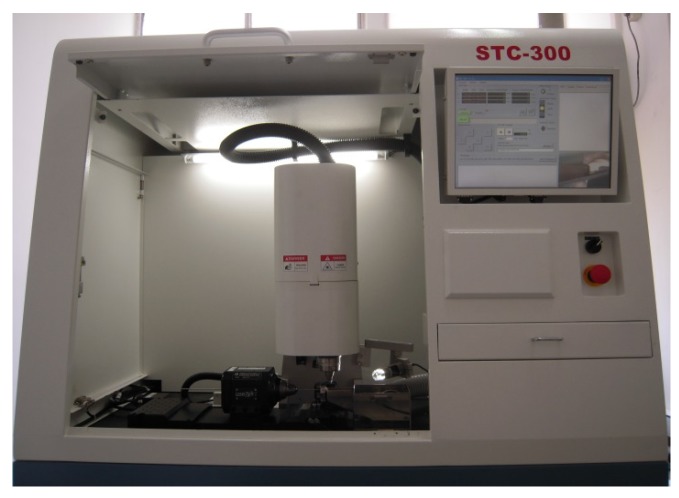
Integrated pulsed fiber laser module.

A laser module consisting of a Rofin 100 W pulsed fiber laser, an Aerotech linear *X*–*Y* motor stage, and a *Z*-direction server motor were assembled together and integrated (Figure 5). The accuracy of the motor stage was ±2 μm in the axial direction and ±25 arc-seconds in the circumferential direction. The linear motion and rotation of the hypotube were provided by the linear *X*–*Y* motor stage, while the distance between the laser source and hypotube surface was controlled by the *Z*-direction server motor to achieve the optimal focal position.

**Figure 6:**
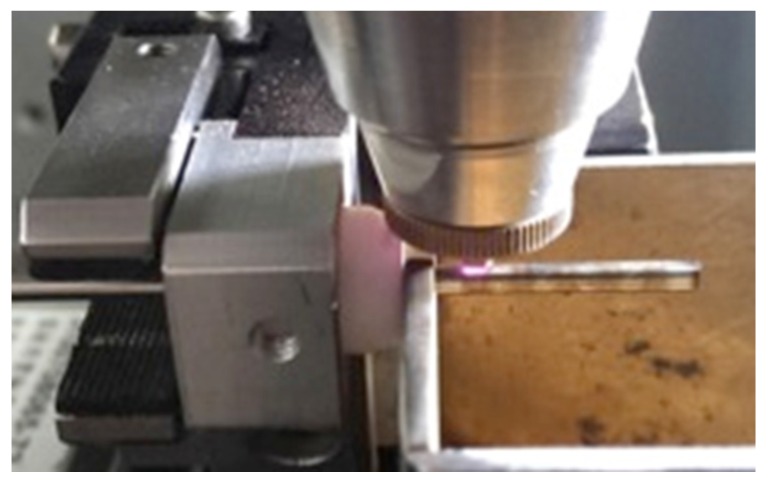
Coded design pattern of the spherical occlusion device cut into a 2.0 mm hypotube by laser.

During the manufacture of the spherical occlusion device, a 2D drawing was first sketched on the *X-Y* plane and then coded into the 3D cylindrical coordinate system by wrapping the 2D sketch around a target cylinder. The device design pattern was cut into a seamless 2.0 mm hypotube based on the coded geometry uploaded to the laser module (Figure 6). Position Synchronized Output (PSO) was the control algorithm used to coordinate the linear *X*–*Y* motor stage with the timing of laser firing. The laser cutting quality and laser output efficiency were greatly enhanced by the PSO algorithm. The Aerotech A3200 stage controller (Aerotech, Inc., Pittsburgh, PA, USA) was able to perform up to 32 axes of synchronized motion control and thus accomplish sophisticated laser cutting patterns.

### 2.5. Heat Treatment and Surface Finishing

The spherical occlusion device is able to self-expand to a target diameter when released from a catheter due to the super-elasticity of nitinol. In order to create such super-elastic behavior, special heat treatment was used after laser cutting to gradually shape the device size or contour to the desired levels and remove residual stresses resulting from expansions. During this heat-treatment process, the two most critical factors are time and temperature. When the processing time is too short, residual stresses from each expansion cannot be completely relieved. As a result, the spherical occlusion device may not be heat-treated to the desired size or shape, increasing the potential risk of fracture during the next expansion or clinical use. When the processing time is too long, heat treatment becomes inefficient and the properties of the nitinol may be degraded during the process. High temperature could lead to the formation of a TiO_2_ oxide layer on the device surface in a short time, which would require extra surface finishing at a later stage.

**Figure 7:**
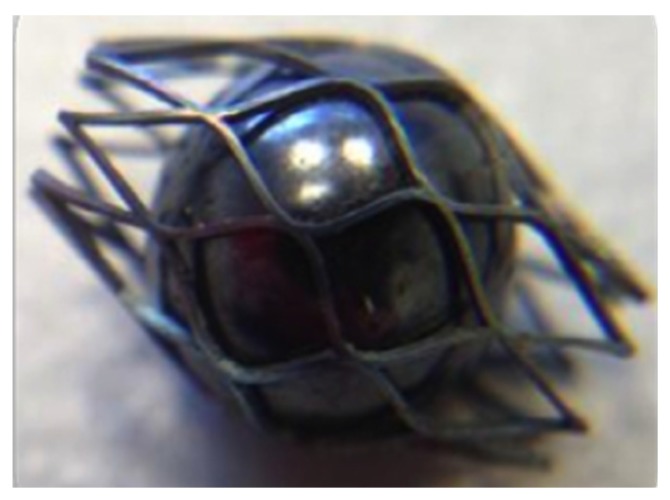
Expansion and shaping of the spherical occlusion device by inserting a steel ball.

In this study, the spherical occlusion device was expanded and shaped progressively by inserting steel balls of increasing diameters and then heat-treated in a salt bath at an elevated temperature for a certain time after each expansion (Figure 7). In order to help select the appropriate heat-treatment time and temperature, experiments were conducted, and the device shape setting rate *R_setting_* was defined as:

(5) $$\;R_{setting}=\frac{OD_{device}}{\Phi_{ball}+2\times t}$$

where *OD_device_* is the outer diameter of the spherical occlusion device after heat treatment, φ*_ball_* is the diameter of the steel ball, and *t* is the thickness of the spherical occlusion device.

The high temperatures associated with laser cutting and heat treatment may create spatter, oxide layers, and other debris that must be removed with further processing. Surface finishing was accomplished in two major steps: sand-blasting and electro-polishing. Sand-blasting was first applied with aluminum oxide particles (37–44 μm) sprayed at a pressure of 2 kg/cm^2^ for one minute to remove large debris, followed by electro-polishing to further improve the surface condition. The electro-polishing solution was a mixture of 79% (volume) of acetic acid and 21% (volume) of perchloric acid. The anode and cathode were made of a stainless steel flake and titanium wire, respectively. The electro-polishing process was conducted at room temperature in two stages: one of 7.7 volts for 60 s and then one of 11.3 volts for another 60 s.

## 3. Results and Discussion

### 3.1. Novel Spherical Occlusion Device Design

**Figure 8:**
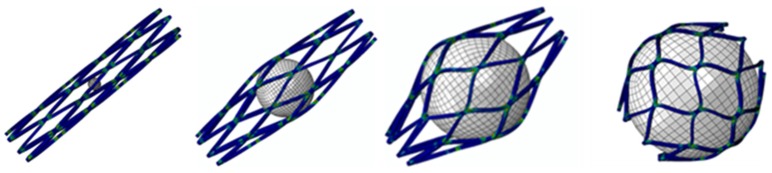
Contour plots of strain distribution during a series of spherical expansions.

The nitinol spherical occlusion device was made in an alternating series of expansion and heat-treatment procedures as the device was progressively formed into its final spherical shape and diameter of 5.2 mm. Figure 8 shows the contour plots of the strain distribution during the multiple spherical expansions. During each expansion, the maximum strain occurred at the inner surface of the most curved regions, while there was little to no strain at the adjacent straight regions. The repeated heat treatment after each expansion relieved all stresses, so the entire spherical occlusion device returned to its stress-free state. The deformed geometry of the device was then extracted for the next expansion until the final diameter of 5.2 mm was reached.

**Figure 9:**
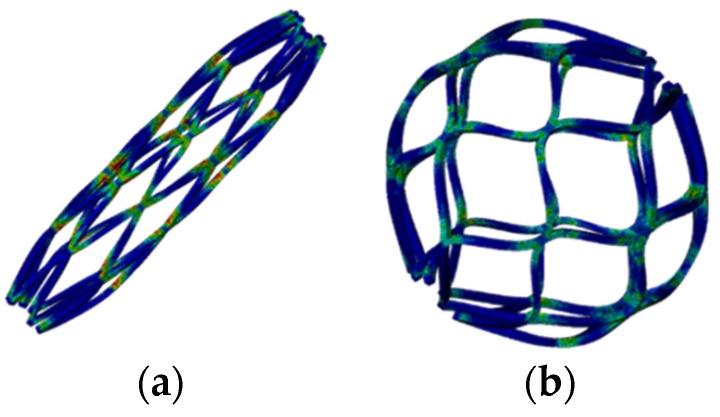
Contour plots of strain distribution (**a**) at crimping and (**b**) after release into an artery.

After the completion of multiple expansions and heat treatments, the spherical occlusion device was crimped to the size of 1.6 mm and constrained inside a catheter. It was then released from the catheter to allow for spring-back to the target diameter of 5.2 mm. Figure 9 shows the contour plots of the strain distribution at crimping and after release into an artery. The crimping appeared to be the most critical stage of all, as the highest strain was recorded during this step (Table 1). Overall, our spherical occlusion device is able to withstand large deformations and then self-expand to the target shape and size as designed.

**Table 1:** Maximum strain occurring during the manufacturing and deployment procedures.

Manufacturing/Deployment	Maximum Strain
Expand to 2.0 mm	0.1%
Expand to 3.0 mm	4.6%
Expand to 4.0 mm	6.5%
Expand to 5.2 mm	9.1%
Crimp to 1.6 mm	10.0%
Release to 5.2 mm	0.3%

### 3.2. Occlusion Effects

**Figure 10:**
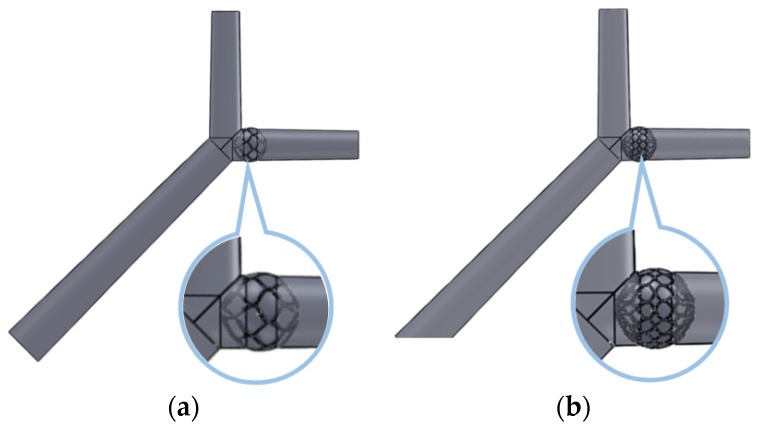
Spherical occlusion device placed in the right hepatic artery near the branch with (**a**) lower and (**b**) higher occlusion densities.

**Figure 11:**
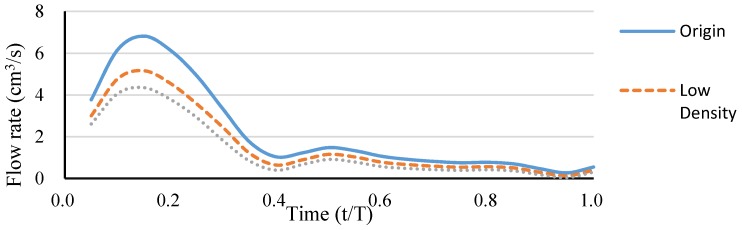
Blood flow rate reduction as a function of time in a single pulse in the right hepatic artery.

A simple hepatic artery bifurcation model was constructed to evaluate the effects of the presence of the spherical occlusion device on the flow rate change. This bifurcation model represented a common hepatic artery branching into smaller right and left hepatic arteries. The spherical occlusion device was placed in the upstream location of the right hepatic artery near the branch, with two different occlusion densities evaluated for the flow change (Figure 10). The device on the right in Figure 10 had a metal density (27.2%) twice that of its counterpart on the left (13.6%). Simulation results showed that the presence of the spherical occlusion device at the indicated location had significant effects on the flow rate. Figure 11 shows the reduction in blood flow rate as a function of time in a single pulse in the right hepatic artery. When the spherical occlusion device with a higher density was deployed in the upstream location of the right hepatic artery, the average blood flow rate in a single pulse was reduced by almost 50%. The lower-density device reduced the flow rate by 30% on average. The flow rate reduction in the right hepatic artery implies that more blood was re-routed to the left hepatic artery due to mass conservation. It should be noted that some flow is still required in order to carry chemotherapy or radioactive drugs to the downstream location of the right hepatic artery. The flow rate can be tailored to a desired level, depending on the density of the spherical occlusion device designed.

### 3.3. Prototype Laser Cutting

**Figure 12:**
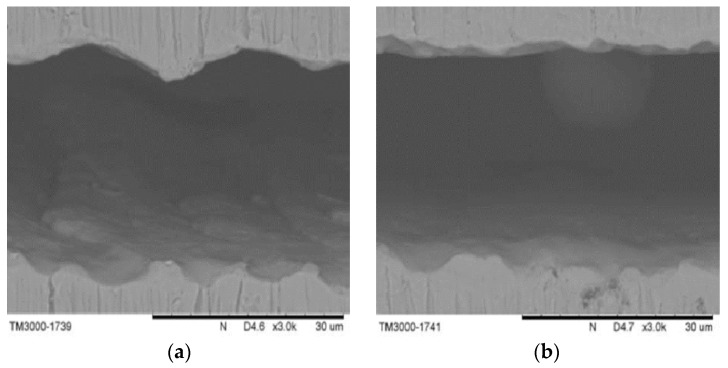
Scanning electron microscope (SEM) pictures of (**a**) poor and (**b**) good laser cutting quality.

The quality of the laser cutting is governed by several laser parameters, such as pulse repetition rate, focal position, and assisted gas pressure. The pulse repetition rate is an important laser parameter related to material removal and surface roughness. A higher pulse repetition rate corresponds to more effective material removal and better surface cutting quality. Scanning Electron Microscope (SEM) images showed that different pulse repetition rates led to two different cutting results (Figure 12). A higher pulse repetition rate corresponded to a better surface cutting quality, whereas poor cutting could cause energy to accumulate and form larger heat affected zones. The focal position is another important laser parameter. The laser cutting outcome is significantly improved by an appropriate focal position.

**Figure 13:**
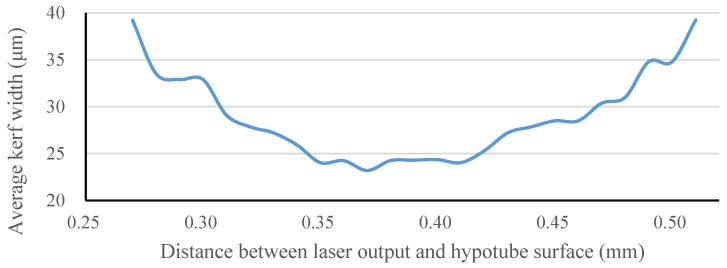
Kerf width *vs.* *Z*-direction distance between the laser source and hypotube surface.

The focal position of our laser module was determined by measuring the kerf width on the hypotube while changing the *Z*-direction distance between the laser source and hypotube surface. Optical microscopy was used to measure the kerf width and observe the surface conditions of each prototype. The relationship of the kerf width to the *Z*-direction distance between the laser source and hypotube surface is shown in Figure 13. To achieve the smallest kerf width, the laser beam was focused on the hypotube surface precisely, in an attempt to find the optimal focal depth. When the *Z*-direction distance was between 0.27 and 0.51 mm, the laser beam was able to penetrate through the hypotube, resulting in successful cutting within a focal-depth range of 0.24 mm in the *Z*-direction. At the *Z*-direction distance of 0.37 mm, the kerf width could be reduced to 23.2 μm.

### 3.4. Heat Treatment and Surface Finishing

A temperature of 500 °C was first used to establish the baseline to help identify the appropriate heat-treatment temperatures for our spherical occlusion device [21]. Experiments were conducted at various heat-treatment temperatures (475–525 °C) with various processing times (150–300 s) to find the best heat-treatment parameter combinations and their effects on the shape-setting rate of the spherical occlusion device.

**Figure 14:**
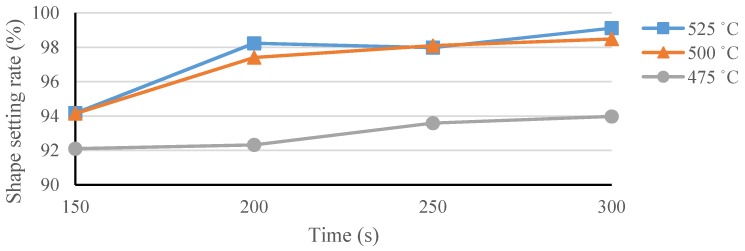
Shape setting rate of the spherical occlusion device.

The shape setting rate results of the spherical occlusion device are shown in Figure 14 and the experimental data are presented in Table 2. It was found that the heat-treatment temperature of 475 °C was not able to yield a shape setting rate greater than 95%, regardless of the time spent. In contrast, both 500 and 525 °C heat-treatment temperatures, combined with a processing time longer than 200 s, were able to achieve a shape setting rate of close to 100%. Since a lower heat-treatment temperature with shorter processing time had less negative effects on the device properties, a heat-treatment temperature of 500 °C and a processing time of 200 s were selected as the heat-treatment parameters for making the prototype of the spherical occlusion device.

**Table 2:** Experimental data of the measured shape setting rate.

Temperature (°C)	Time (s)	Shape Setting Rate (%)			
		Sample 1	Sample 2	Sample 3	Average
475	150	93.11	91.02	92.16	92.10
	200	92.80	93.22	90.93	92.32
	250	93.10	93.59	94.08	93.59
	300	94.28	93.76	93.87	93.97
500	150	95.10	94.20	93.12	94.14
	200	97.10	96.44	98.67	97.40
	250	98.19	97.87	98.23	98.09
	300	96.97	98.93	99.55	98.48
525	150	95.12	95.10	92.28	94.17
	200	98.40	98.96	97.33	98.23
	250	98.45	97.97	97.53	97.98
	300	98.59	99.46	99.30	99.12

**Figure 15:**
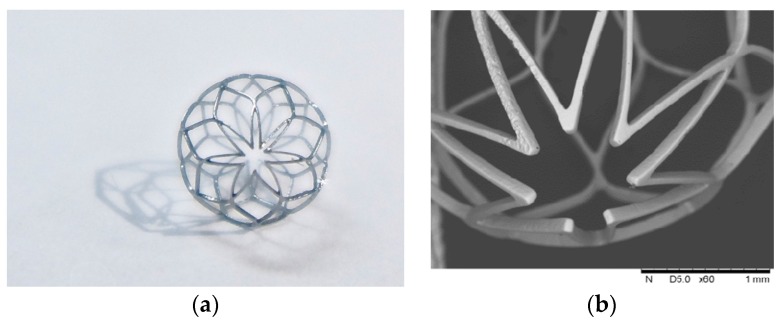
(**a**) First prototype in the world of the spherical occlusion device and (**b**) its SEM image.

After laser cutting, the spherical occlusion device was expanded gradually to the desired size/contour and heat-treated multiple times in a salt bath at 500 °C for 200 s. The device surface was then sand-blasted and electro-polished to achieve a mirror-like surface finish. Figure 15 presents the world’s first prototype of the spherical occlusion device for the demonstration of our novel design concept.

## 4. Conclusions

In this paper, we propose a novel nitinol “spherical occlusion device” concept, the first of its kind in the world, which provides a new option for future liver cancer treatment. Our proposed spherical occlusion device can reduce the blood flow to cancer cells when deployed in the upstream hepatic artery supplying blood to the liver. We applied the parametric design scheme to the design of the spherical occlusion device and integrated it with the developed computational models to evaluate the mechanical and hemodynamic performances of the spherical occlusion device during manufacturing and deployment. Simulation results showed that the presence of the spherical occlusion device with density ranging from 14%–27% had significant effects on the flow rate. When such spherical occlusion devices were deployed at the upstream location of the right hepatic artery, the average blood flow rate was reduced by 30%–50%. This flow rate can be tailored to a desired level by adjusting the densities of the spherical occlusion device. A pulsed fiber laser, integrated with a precision *X*–*Y* motor stage, was used to make the first prototype of the spherical occlusion device for the demonstration of our novel design concept.
